# Polatuzumab Vedotin Enhances Intratumoral T‐Cell Infiltration and Demonstrates Combinatorial Efficacy With Anti‐CD20/CD3 Bispecific Antibody in a Syngeneic B‐Cell Malignancy Mouse Model

**DOI:** 10.1002/jha2.70349

**Published:** 2026-07-08

**Authors:** Mayu Tomita, Sei Shu, Natsumi Kawasaki, Satoko Takagi, Shigeki Yoshiura, Yoriko Yamashita‐Kashima

**Affiliations:** ^1^ Product Research Department Chugai Pharmaceutical Co. Ltd. Yokohama Japan

**Keywords:** anti‐CD20/CD3 bispecific antibody, diffuse large B‐cell lymphoma (DLBCL), mosunetuzumab, T cells, polatuzumab vedotin, tumor microenvironment

## Abstract

**Introduction:**

Polatuzumab vedotin (Pola) is an anti‐CD79b antibody‐drug conjugate. Clinical evidence supports the combination efficacy of Pola with mosunetuzumab or glofitamab, both anti‐CD20/CD3 bispecific antibodies (anti‐CD20/CD3 BsAbs). However, the influence of Pola on the tumor microenvironment and its impact on the efficacy of anti‐CD20/CD3 BsAb remains unclear.

**Results:**

In a syngeneic mouse model, Pola significantly increased tumor‐infiltrating T cells and innate immune cells. Moreover, Pola also upregulated the levels of cytokine and chemokine involved in T‐cell infiltration. The Pola + anti‐CD20/CD3 BsAb combination showed superior anti‐tumor efficacy versus each monotherapy.

**Conclusion:**

These findings suggest Pola enhances immune cell infiltration into tumors and could enhance the efficacy of anti‐CD20/CD3 BsAb.

**Trial Registration:**

The authors have confirmed clinical trial registration is not needed for this submission

## 1 Introduction

Polatuzumab vedotin (Pola), an antibody‐drug conjugate comprising an anti‐CD79b antibody linked to monomethyl auristatin E (MMAE), has been approved in multiple countries for the treatment of diffuse large B‐cell lymphoma (DLBCL) [1, 2]. Meanwhile, multiple anti‐CD20/CD3 bispecific antibodies (anti‐CD20/CD3 BsAbs), such as mosunetuzumab (Mosun) and glofitamab (Glofit), are either approved or in development. These agents activate T cells to eliminate tumor cells by simultaneously binding to CD3 on T cells and CD20 on B cells [3, 4]. Clinical evidence has demonstrated the safety and efficacy of Mosun or Glofit combined with Pola in DLBCL [5, 6].

Our prior nonclinical studies revealed that Pola promotes infiltration of innate immune cells, particularly macrophages (MΦs) and natural killer cells (NK cells) into the tumor microenvironment, thereby contributing to its anti‐tumor effects [7]. However, Pola's impact on the adaptive immune system in tumors remains unclear both in clinical and nonclinical settings.

In the present study, we investigated Pola's effects on the tumor microenvironment, including T cells, and assessed the combination efficacy with anti‐CD20/CD3 BsAb utilizing a syngeneic mouse model.

## 2 Methods

### 2.1 Reagents

Pola was obtained from Chugai Pharmaceutical and anti‐murine CD20 (5D2)‐anti‐murine CD3 (2C11) bispecific antibody (anti‐mCD20/mCD3 BsAb) was obtained from Genentech. Human immunoglobulin G (hIgG) and Ultra‐LEAF Purified Mouse IgG2a, κ Isotype Ctrl Antibody (mIgG) were purchased from MP Biomedicals and BioLegend, respectively.

### 2.2 Establishment of a New Cell Line and Cell Cultures

L1210‐hCD79b‐9 cells (Pola‐sensitive B‐lymphocytic leukemia cells, expressing human‐mouse chimeric CD79b containing the Pola‐binding domain) were established as previously described [7]. A plasmid vector containing the murine CD20 gene (*mMs4a1*) was purchased from VectorBuilder. L1210‐hCD79b‐9 cells were transformed with this plasmid by electroporation. Following single‐cell cloning, one clone was established as the L1210‐hCD79b‐mCD20‐8 cell line.

These cells were cultured in Dulbecco's Modified Eagle's Medium High Glucose (Sigma) supplemented with 10% horse serum (Thermo Fisher Scientific) and 1 mM sodium pyruvate (Thermo Fisher Scientific). All cells were cultured at 37°C under 5% CO_2_.

### 2.3 Animals and In Vivo Experiments

All animal experiments were reviewed and approved by the Institutional Animal Care and Use Committee at Chugai Pharmaceutical Co. Ltd., an institute accredited by AAALAC International. Practices at our institute conform to those stipulated in the *Guide for the Care and Use of Laboratory Animals* published by the Institute for Laboratory Animal Research.

DBA/2NCrl mice (female, 7–8‐week old) were obtained from The Jackson Laboratory Japan, Inc. Mice were inoculated subcutaneously with 5 × 10^6^ L1210‐hCD79b‐mCD20‐8 cells.

After tumor engraftment, mice were randomized into treatment groups and received intravenous administration of the following agents on Day 1: 2 mg/kg Pola, 2 mg/kg hIgG, 0.3 mg/kg anti‐mCD20/mCD3 BsAb, and/or 0.3 mg/kg mIgG. To evaluate the anti‐tumor activity of the test agents, tumor volume was assessed as described previously [8].

### 2.4 Analysis of Cell Surface Molecule Expression and Immunophenotyping by Flow Cytometry

In vivo tumor samples were collected on Day 4 after Pola administration and digested using the Tumor Dissociation Kit, mouse (Miltenyi Biotec). Cells prepared either in vitro or in vivo were stained with the antibodies described in the Supporting Information.

Stained cells were analyzed using BD LSRFortessa X‐20 (BD Biosciences) and FlowJo v10 software (BD Biosciences).

### 2.5 Cell Viability Assay

Cells were incubated with Pola at varying concentrations. After 3 days, CellTiter‐Glo 3D Cell Viability Assay reagent (Promega) was added to each well, and luminescence was measured using a Varioskan LUX Multimode Microplate Reader (Thermo Fisher Scientific). For each drug concentration test, cell viability was calculated as a percentage of the control value.

### 2.6 T‐Cell‐Dependent Cellular Cytotoxicity (TDCC) Assay

Mouse splenocytes were prepared by dissociating spleens collected from DBA/2NCrl mice into single cells, which were then cryopreserved. Mouse T cells were purified from the splenocytes using an EasySep Mouse T Cell Isolation Kit (STEMCELL Technologies). Target cells were labeled using CellTrace Violet Cell Proliferation Kit (Thermo Fisher Scientific) and seeded, then anti‐mCD20/mCD3 BsAb and effector T cells were added at an appropriate effector/target ratio (ET ratio). After incubation, dead cells were stained using Fixable Viability Dye (FVD; Thermo Fisher Scientific). Target‐specific cell killing was detected by FVD staining of target cells using the flow cytometer (BD LSRFortessa X‐20).

### 2.7 In Vitro Damage‐Associated Molecular Pattern (DAMP) Analysis

L1210‐hCD79b‐mCD20‐8 cells were incubated with MMAE (Selleck) at varying concentrations. After 3 days, High Mobility Group Box 1 (HMGB1) was assessed by using Lumit HMGB1 Human/Mouse Immunoassay (Promega). Total fluorescence intensity was determined with a Varioskan LUX Multimode Microplate Reader (Thermo Fisher Scientific).

### 2.8 Luminex Mouse Cytokine Multiplex Assay

ProcartaPlex Mouse 6‐plex Assay Kit (Thermo Fisher Scientific) for Luminex (Thermo Fisher Scientific) was used to measure the following cytokines in tumor lysates according to the manufacturer's protocol: IP‐10 (CXCL10), IFN‐γ, RANTES (CCL5), and MCP‐1 (CCL2). A standard curve was generated by preparing a serial dilution of standards in the presence of a drug‐untreated tumor, and concentrations in the test samples were calculated from this standard curve.

### 2.9 Statistical Analysis

Student's *t*‐test was used for two‐group comparisons and Dunnett's test was used for multiple comparisons with the control group. Pearson's correlation test was used to determine the correlation between two variables. For tumor syngeneic experiments, *p*‐values were adjusted for the Wilcoxon rank sum test by the Holm–Bonferroni method. Statistical analyses were performed with statistical software packages JMP v17.2 (SAS Institute).

## 3 Results and Discussion

### 3.1 Pola Promotes Infiltration of T Cells and Other Immune Cells

We previously established L1210‐hCD79b‐9 cells by expressing human‐mouse chimeric CD79b in a mouse B‐cell line to evaluate Pola efficacy in immunocompetent mice [7]. Since this cell line lacked mouse CD20 (Figure S1A), we further established L1210‐hCD79b‐mCD20‐8 cells expressing mouse CD20 to evaluate anti‐mCD20/mCD3 BsAb. L1210‐hCD79b‐mCD20‐8 cells were characterized for chimeric CD79b and mouse CD20 expression (Figure S1B,C), were sensitive to Pola, and showed Tcell‐dependent tumor cell killing in the presence of mouse anti‐mCD20/mCD3 BsAb in vitro (Figure S1D,E).

**FIGURE 1:**
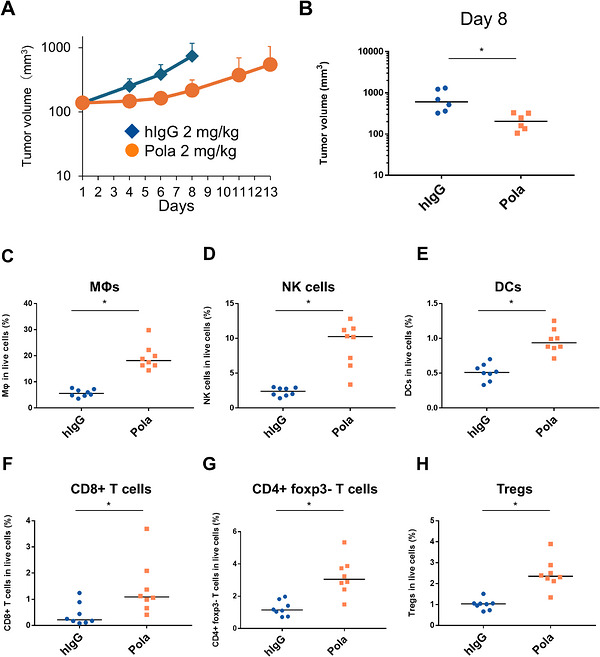
Pola increased infiltration of immune cells into tumors. (A) In vivo L1210‐hCD79b‐mCD20‐8 tumor growth curves from Day 1 to Day 13 after administration of 2 mg/kg Pola (*n*  =  6). The data represent the mean + SD (B) Individual tumor volumes in each group at Day 8 (*n*  =  6). *p < 0.05 by Wilcoxon rank sum test. In dot plots, horizontal bars represent median values. (C–H) Percentages of (C) MΦs, (D) NK cells, (E) DCs, (F) CD8+ T cells, (G) CD4+Foxp3‐ T cells, and (H) Tregs among live cells at Day 4 after administration of 2 mg/kg Pola (*n* = 8). *p < 0.05 by Student's *t*‐test. In dot plots, horizontal bars represent median values.

In the L1210‐hCD79b‐mCD20‐8 syngeneic model, the Pola 2 mg/kg treatment group showed significant anti‐tumor efficacy compared to the control group (Figure 1A,B). To investigate the impact of Pola on diverse immune cell populations within the tumor, we assessed intratumoral immune cells by flow cytometry. These analyses revealed Pola‐mediated infiltration of dendritic cells (DCs), CD8+ T cells, CD4+Foxp3‐ helper T cells, and CD4+Foxp3+ regulatory T cells (Tregs), in addition to MΦs and NK cells as previously reported [7] (Figure 1C–H).

### 3.2 Pola Treatment Increases Multiple Factors Involved in T‐Cell Infiltration Within Tumors

Our previous reports indicate that Pola‐induced DAMP release enhances innate immune cell infiltration into tumors [7]. Previous studies have shown that immunogenic cell death (ICD) triggers release of DAMPs (e.g., HMGB1) and cytokines/chemokines, such as IP‐10 (CXCL10) and MCP‐1 (CCL2), leading to T‐cell recruitment either directly or indirectly through DCs [9, 10].

**FIGURE 2:**
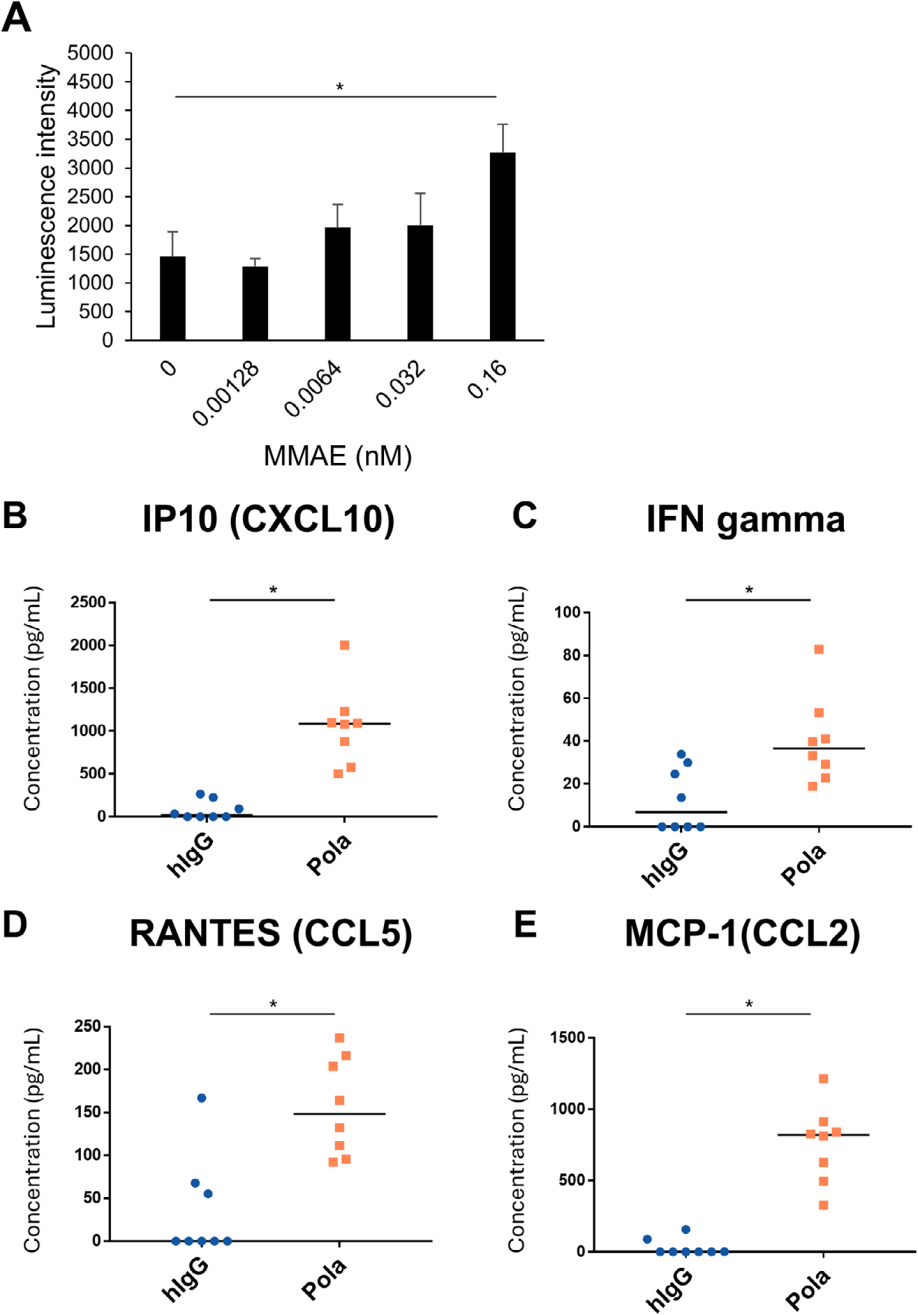
Pola increases intratumoral ICD‐related factors involved in T‐cell recruitment. (A) HMGB1 released from L1210‐hCD79b‐mCD20‐8 cells 3 days after MMAE treatment in vitro (*n* = 3). The data represent the mean + SD; *p < 0.05 by Dunnett's test compared to MMAE 0 nM. (B–E) Concentrations of (B) IP‐10 (CXCL10), (C) IFN‐γ, (D) RANTES (CCL5), and (E) MCP‐1 (CCL2) in tumors at Day 4 after 2 mg/kg Pola administration (*n* = 8). *p < 0.05 by Student's *t*‐test. In dot plots, horizontal bars represent median values.

To investigate the mechanisms of enhanced immune cell infiltration, particularly by T cells (the effectors of anti‐CD20/CD3 BsAb), we quantified ICD‐related factors after Pola treatment in the L1210‐hCD79b‐mCD20‐8 model. Our previous reports have shown that DAMPs released by Pola are mediated by MMAE in human DLBCL cells [7]. Also, in the L1210‐hCD79b‐mCD20‐8 cells, MMAE enhanced HMGB1 release (Figure 2A). In addition, Pola treatment significantly increased intratumoral IP‐10 (CXCL10), IFN‐γ, RANTES (CCL5), and MCP‐1 (CCL2) levels compared to Ctrl IgG (Figure 2B–E). These results suggest that Pola elevates ICD‐associated and T cell‐infiltrating factors in tumors, thereby promoting immune cell infiltration in association with MMAE‐induced cell death.

### 3.3 Pola Shows Combinatorial Efficacy With Anti‐mCD20/mCD3 BsAb

Mosun exhibits enhanced efficacy in immunologically “hot” DLBCL tumors characterized by multiple immune cell infiltration, particularly those rich in T cells, compared with those classified as “cold” [11]. In addition, the anti‐mCD20/mCD3 BsAb showed ET ratio‐dependent killing in vitro (Figure S2A).

**FIGURE 3:**
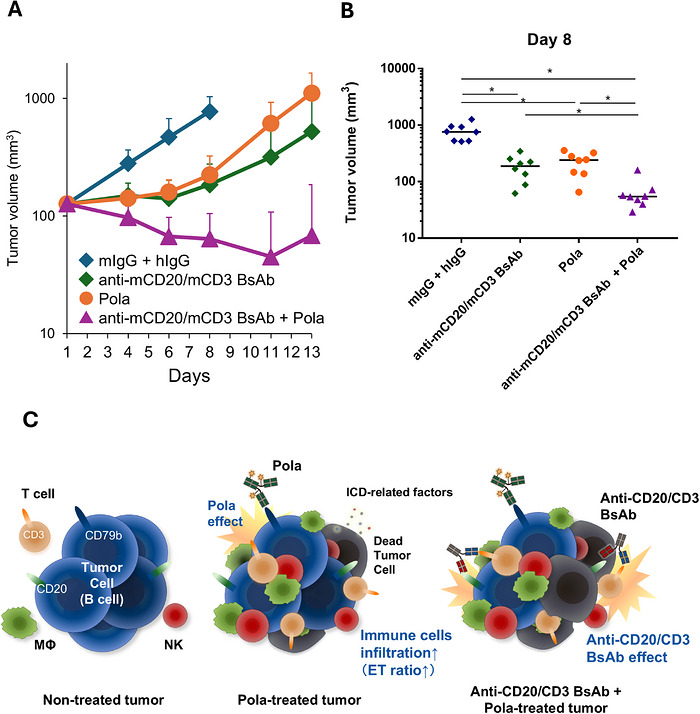
Pola and anti‐mCD20/mCD3 BsAb demonstrate combination effects in vivo. (A) In vivo L1210‐hCD79b‐mCD20‐8 tumor growth curves from Day 1 to Day 13 after administration of 2 mg/kg Pola and/or 0.3 mg/kg anti‐mCD20/mCD3 BsAb (*n*  =  8). The data represent the mean + SD. (B) Individual tumor volumes in each group at Day 8 (*n*  =  8). *p < 0.05 by Wilcoxon rank sum test adjusted by the Holm–Bonferroni method. In dot plots, horizontal bars represent median values. (C) Schematic diagram illustrating the relationship between the anti‐tumor effect of anti‐mCD20/mCD3 BsAb + Pola and the tumor microenvironment.

Based on the findings of Pola‐mediated enhancement of immune infiltration, we hypothesized that Pola enhances the therapeutic efficacy of anti‐mCD20/mCD3 BsAb in the L1210‐hCD79b‐mCD20‐8 syngeneic mouse model. Therefore, we evaluated anti‐mCD20/mCD3 BsAb + Pola combination therapy in mice. In the L1210‐hCD79b‐mCD20‐8 syngeneic mouse model, combining Pola with the anti‐mCD20/mCD3 BsAb showed significantly enhanced anti‐tumor efficacy compared to either monotherapy on Day 8 (Figure 3A,B). Our previous studies suggested that Pola enhances bispecific antibody efficacy through CD20 upregulation in humanized mouse models under immunodeficient conditions [12]; however, substantial CD20 upregulation was not observed in L1210‐hCD79b‐mCD20‐8 cells (Figure S2B).

Pola showed a combinational effect with anti‐mCD20/mCD3 BsAb in this model without upregulation of CD20 expression, suggesting mechanisms different from those previously reported ones [12, 13]. Our results also provide supporting evidence that Pola not only directly induces apoptosis of tumor cells but also modulates the tumor microenvironment, thereby promoting infiltration of a broad range of immune cells, including T cells. Pola‐induced T cell infiltration may represent one of the mechanisms underlying the combinational effect (Figure 3C). Future studies using multiple animal models or clinical specimens are warranted to further validate these findings.

## 4 Conclusion

Pola treatment enhanced immune cell infiltration, particularly by T cells, and demonstrated superior anti‐tumor efficacy when combined with anti‐CD20/CD3 BsAb compared to monotherapies.

## Author Contributions

**Mayu Tomita**: conceptualization, data curation, investigation, methodology, writing – original draft, writing – review and editing. **Sei Shu**: conceptualization, data curation, investigation, methodology, writing – review and editing. **Natsumi Kawasaki**: data curation, investigation, methodology, writing – review and editing. **Satoko Takagi**: data curation, investigation, methodology, writing – review and editing. **Shigeki Yoshiura**: conceptualization, supervision, methodology, project administration, writing – review and editing. **Yoriko Yamashita‐Kashima**: conceptualization, supervision, methodology, project administration, writing – original draft, writing – review and editing.

## Funding

The authors have nothing to report.

## Ethics Statement

This study does not qualify as clinical research. All animal experiments were reviewed and approved by the Institutional Animal Care and Use Committee at Chugai Pharmaceutical Co. Ltd., an institute accredited by AAALAC International.

## Consent

The authors have nothing to report.

## Conflicts of Interest

All authors are employees of Chugai Pharmaceutical Co. Ltd.

## Supporting information



Supporting Information File 1: jha270349‐sup‐0001‐SuppMat.docx

## Data Availability

All data underlying this study are available from the corresponding author upon reasonable request.
