# Supplementary material for: Polatuzumab Vedotin Enhances Intratumoral T‐Cell Infiltration and Demonstrates Combinatorial Efficacy With Anti‐CD20/CD3 Bispecific Antibody in a Syngeneic B‐Cell Malignancy Mouse Model
Source: EJHaem. 2026 Jul 8;7(4):e70349. doi: 10.1002/jha2.70349 (PMC13343289; doi:10.1002/jha2.70349)
Supplement: Supplementary file 1 — Supporting Information File 1: jha270349‐sup‐0001‐SuppMat.docx [file JHA2-7-e70349-s001.docx]

Supplementary Methods

Antibodies for Flow Cytometry Staining

Cells were stained with the following antibodies: Anti-CD20 (clone: SA275A11, BioLegend), Anti-CD79b (clone: SN8 [identical to the antibody portion of Pola], BD Biosciences), Anti-CD8 (clone: KT15, MBL Life Science), Anti-Foxp3 (clone: FJK-16s, Invitrogen), Anti-CD45 (clone: 30-F11, BioLegend or BD Biosciences), Anti-CD4 (clone: GK1.5, BD Biosciences), Anti-CD3 (clone: 17A2, BD Biosciences), Anti-Gr-1 (clone: RB6-8C5, BioLegend), Anti-CD11b (clone: M1/70, BD Biosciences), Anti-CD11c (clone: N418, BioLegend), Anti-NKp46 (clone: 29A1.4, BioLegend), Anti-CD19 (clone: 6D5, BioLegend), Anti-F4/80 (clone: T45-2342, BD Biosciences), and Anti-I-A/I-E (clone: 2G9, BD Biosciences). IgG1κ (clone: X40, BD Biosciences) and IgG2bκ (clone: RTK4530, BioLegend) were used as isotype controls.

# Supplementary Figures


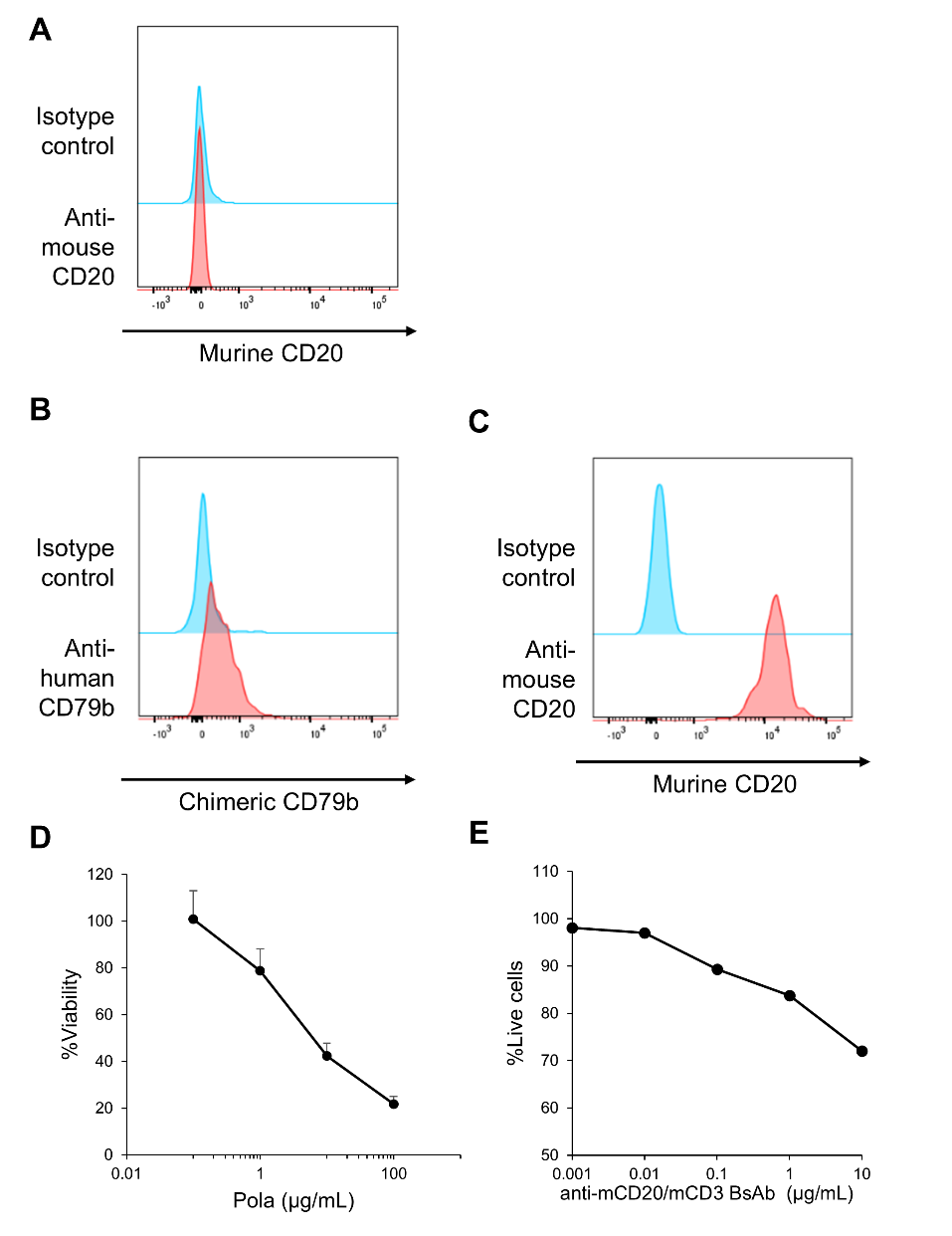

**Fig. S1. In vitro validation of L1210-hCD79b-mCD20-8 cell line.**

**(A)** Surface expression of murine CD20 on L1210-hCD79b-9 cells. **(B)** Surface expression of Chimeric CD79b on L1210-hCD79b-mCD20-8 cells. **(C)** Surface expression of murine CD20 on L1210-hCD79b-mCD20-8 cells. **(D)** Sensitivity to Pola at the indicated concentrations was assessed in L1210-hCD79b-mCD20-8 cells (n = 3). The data represent the mean + SD. **(E)** Sensitivity to anti-mCD20/mCD3 BsAb at the indicated concentrations was assessed in L1210-hCD79b-mCD20-8 cells at an ET ratio of 2.5 (n = 2). The data represent the mean.


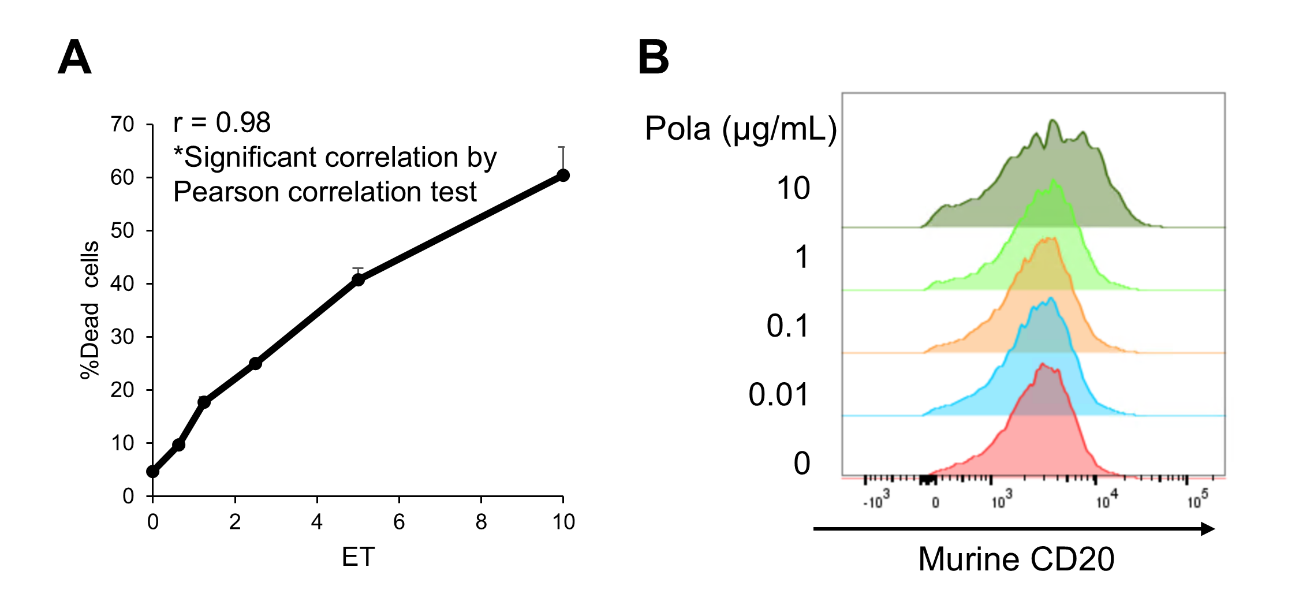


**Fig. S2. ET ratio-dependent efficacy of anti-mCD20/mCD3 BsAb and Pola-mediated modulation of CD20 expression in L1210-hCD79b-mCD20-8 cells in vitro**

(A) In vitro ET ratio-dependent dead cell percentage of L1210-hCD79b-mCD20-8 cells treated with 10 μg/mL anti-mCD20/mCD3 BsAb (n = 3). The data represent the mean + SD. * P < 0.05 by Pearson's correlation analysis. (B) In vitro surface expression of murine CD20 on L1210-hCD79b-mCD20-8 cells at various Pola concentrations.
